# Supervised machine learning enables non-invasive lesion characterization in primary prostate cancer with [^68^Ga]Ga-PSMA-11 PET/MRI

**DOI:** 10.1007/s00259-020-05140-y

**Published:** 2020-12-19

**Authors:** L. Papp, C. P. Spielvogel, B. Grubmüller, M. Grahovac, D. Krajnc, B. Ecsedi, R. A.M. Sareshgi, D. Mohamad, M. Hamboeck, I. Rausch, M. Mitterhauser, W. Wadsak, A. R. Haug, L. Kenner, P. Mazal, M. Susani, S. Hartenbach, P. Baltzer, T. H. Helbich, G. Kramer, S.F. Shariat, T. Beyer, M. Hartenbach, M. Hacker

**Affiliations:** 1 QIMP Team, Center for Medical Physics and Biomedical Engineering, Medical University of Vienna, Vienna, Austria; 2 Department of Biomedical Imaging and Image-guided Therapy, Division of Nuclear Medicine, Medical University of Vienna, Währinger Gürtel 18-20, 1090 Vienna, Austria; 3 Christian Doppler Laboratory for Applied Metabolomics, Vienna, Austria; 4 Department of Urology, Medical University of Vienna, Vienna, Austria; 5 Ludwig Boltzmann Institute Applied Diagnostics, Vienna, Austria; 6 Clinical Institute of Pathology, Medical University of Vienna, Vienna, Austria; 7 HistoConsulting Inc., Ulm, Germany; 8 Department of Biomedical Imaging and Image-guided Therapy, Division of Common General and Pediatric Radiology, Medical University of Vienna, Vienna, Austria

**Keywords:** Prostate cancer, PET/MRI, Radiomics, Machine learning, Lesion risk prediction, Biochemical recurrence prediction, Overall patient risk prediction

## Abstract

**Purpose:**

Risk classification of primary prostate cancer in clinical routine is mainly based on prostate-specific antigen (PSA) levels, Gleason scores from biopsy samples, and tumor-nodes-metastasis (TNM) staging. This study aimed to investigate the diagnostic performance of positron emission tomography/magnetic resonance imaging (PET/MRI) in vivo models for predicting low-vs-high lesion risk (LH) as well as biochemical recurrence (BCR) and overall patient risk (OPR) with machine learning.

**Methods:**

Fifty-two patients who underwent multi-parametric dual-tracer [^18^F]FMC and [^68^Ga]Ga-PSMA-11 PET/MRI as well as radical prostatectomy between 2014 and 2015 were included as part of a single-center pilot to a randomized prospective trial (NCT02659527). Radiomics in combination with ensemble machine learning was applied including the [^68^Ga]Ga-PSMA-11 PET, the apparent diffusion coefficient, and the transverse relaxation time-weighted MRI scans of each patient to establish a low-vs-high risk lesion prediction model (M_LH_). Furthermore, M_BCR_ and M_OPR_ predictive model schemes were built by combining M_LH_, PSA, and clinical stage values of patients. Performance evaluation of the established models was performed with 1000-fold Monte Carlo (MC) cross-validation. Results were additionally compared to conventional [^68^Ga]Ga-PSMA-11 standardized uptake value (SUV) analyses.

**Results:**

The area under the receiver operator characteristic curve (AUC) of the M_LH_ model (0.86) was higher than the AUC of the [^68^Ga]Ga-PSMA-11 SUV_max_ analysis (0.80). MC cross-validation revealed 89% and 91% accuracies with 0.90 and 0.94 AUCs for the M_BCR_ and M_OPR_ models respectively, while standard routine analysis based on PSA, biopsy Gleason score, and TNM staging resulted in 69% and 70% accuracies to predict BCR and OPR respectively.

**Conclusion:**

Our results demonstrate the potential to enhance risk classification in primary prostate cancer patients built on PET/MRI radiomics and machine learning without biopsy sampling.

**Supplementary Information:**

The online version contains supplementary material available at 10.1007/s00259-020-05140-y.

## Introduction

Prostate cancer is the second most common cancer in men worldwide, with 1.3 million new cases diagnosed in 2018 [1, 2]. The worldwide incidence rates significantly increased during the last decade, most likely due to the wider application of prostate-specific antigen (PSA) screening [2]. While the 10-year survival rate of prostate cancer is approximately 90%, advanced or late-stage prostate cancer may be life-threatening, in particular, in metastasized stages of the disease [3].

The 5-year risk stratification in patients with primary prostate cancer is mainly built on clinical stage, PSA, and Gleason scores, derived from invasive biopsy samples [4]. Despite having profound effects on treatment planning and, thus, patient’s quality of life, this approach has a number of limitations [3, 5]. First, Gleason scoring relies on biopsy sampling, hence, can neither help assess the entire prostate nor fully characterize the heterogeneity of any pertinent tumor [6]. In addition, transrectal biopsy sampling has been associated with side-effects, such as haematospermia or haematuria [3]. Second, previously published risk classification systems were reported to have the tendency of incorrectly grading primary prostate cancer [3]. In patients with a high risk score and absent metastatic disease, radical prostatectomy is the treatment-of-choice [7] despite the risk of potential overtreatment [8] and at the same time, a 20–40% chance of biochemical recurrence (BCR) [9, 10].

Combined positron emission tomography/computed tomography (PET/CT) or PET/magnetic resonance imaging (PET/MRI) using radiotracers targeting prostate-specific membrane antigen (PSMA) can help to localize suspicious lesions in the prostate [11, 12]. PSMA-PET in combination with CT has been reported to improve primary tumor localization [13] and the diagnosis of recurrent prostate cancer [14, 15] in patients after radical prostatectomy even at low PSA levels [16]. In contrast, PSMA-PET/MRI was shown to support the diagnosis of intermediate and high-risk patients as well as to detect tumor recurrence [13]. Nevertheless, the diagnosis of primary prostate cancer is still based on core-needle biopsy, with non-invasive imaging playing a role in the visual identification of lesions and/or in image-guidance for biopsy sampling [17, 18].

Recently, radiomics have been argued to add value to the diagnostic pathways and patient management [19]. Various studies have been investigating the correlation of PSMA expression and clinical end-points in prostate cancer patients [14, 15]. Furthermore, radiomics combined with machine learning in MRI [20, 21] as well as in PET/CT [22–24] demonstrated the potential feasibility to establish novel in vivo prediction models for prostate cancer risk assessment.

In light of the potential of combining PET/MR imaging, radiomics and machine learning (ML), the objectives of this study were as follows: (a) to establish and cross-validate prostate lesion low-vs-high risk in vivo ML predictive models built on PET/MRI radiomics, (b) to establish and validate biochemical recurrence and overall patient risk (OPR) models that utilize in vivo ML scores instead of biopsy grades together with PSA and clinical stage, and (c) to compare the above patient risk models to the standard risk stratification.

## Materials and methods

### Patient data

**Fig. 1:**
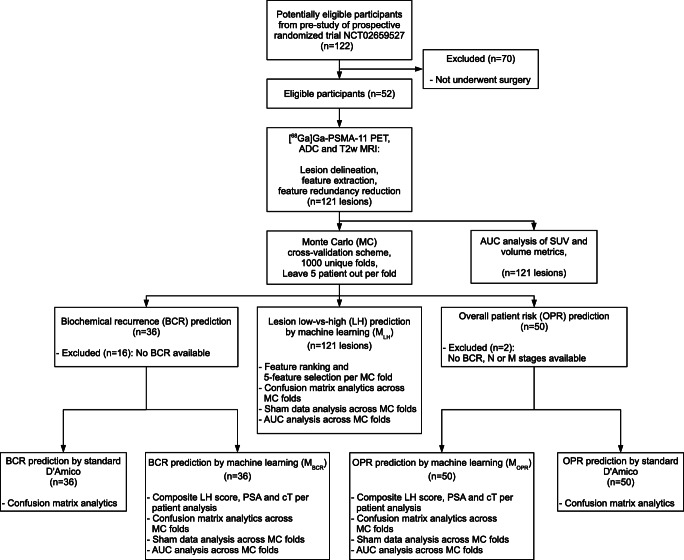
The analysis workflow of the collected dataset. The pre-study of the prospective randomized trial NCT02659527 provided data records of 122 patients between 2014 and 2015. Patients having a dual-tracer positron emission tomography/magnetic resonance imaging (PET/MRI), prostate-specific antigen (PSA) screening, and whole-mount histopathology through undergone surgery were included in the analysis (*n* = 52). Only [^68^Ga]Ga-PSMA-11 PET, apparent diffusion coefficient (ADC), and transverse relaxation time-weighted (T2w) MRI images were selected for radiomic analysis. Overall 121 PET/MRI-positive lesions were delineated from the 52 patients followed by radiomics feature extraction. The 121 lesions underwent prostate specific membrane antigen (PSMA) standardized uptake value (SUV) and volume area under the receiver operator characteristics curve (AUC) analysis. Monte Carlo (MC) cross-validation scheme was utilized to generate patient training and validation sets 1000-times. This MC scheme was utilized to build lesion low-vs-high (LH) prediction models via machine learning (M_LH_). Biochemical recurrence (BCR, *n* = 36) and overall patient risk (OPR, *n* = 50) patient prediction models were built across the same MC folds (M_BCR_ and M_OPR_ respectively). All machine learning models underwent confusion matrix analytics, sham data analysis, and AUC analysis across MC folds. BCR and OPR were also predicted by standard D’Amico score

Patients were selected from the database (*n* = 122) of a mono-centric pilot study to a prospective randomized trial (clinicaltrials.gov NCT02659527) conducted between 2014 and 2015. Fifty-two of the 122 patients underwent surgery; in these patients, PET/MRI, PSA values, pre-operative biopsy results, and post-operative whole-mount histopathology were documented [15] (Table 1). All the 52 patients underwent a dual-tracer, fully integrated PET/MRI scan ([^18^F]FMC and [^68^Ga]Ga-PSMA-11 sequentially). This study, however, only included the [^68^Ga]Ga-PSMA-11 PET image as well as the transverse relaxation time-weighted (T2w) and apparent diffusion coefficient (ADC) MRI sequences in the analysis (Supplement: Table 1). All patients were treated with radical prostatectomy according to guideline recommendations [3]. All surgical specimens were processed according to the institution’s standard pathologic procedures in whole mount sections. Staging and grading were performed according to the UICC TNM classification and WHO/ISUP 2005 system, respectively [25]. The study was approved by the local institutional ethical committee and patients provided their written informed consent. See Fig. 1 for the CONSORT study diagram.

**Table 1:** Characteristics of the 52 patients involved in this study, at the time of radical prostatectomy (RP)

Patient characteristics (*n* = 52)	Value
Age (years), median (IQR)	64 (59–70)
PSA (ng/ml), median (IQR)	7.5 (5.0–13.4)
Pathologic T staging, *n* (ratio)	
2	20 (0.38)
2a	1 (0.02)
2c	2 (0.04)
3a	11 (0.21)
3b	17 (0.33)
4	1 (0.02)
Primary Gleason pattern, *n* (ratio)	
3	18 (0.35)
4	31 (0.6)
5	3 (0.05)
Secondary Gleason pattern, *n* (ratio)	
3	16 (0.31)
4	26 (0.5)
5	10 (0.19)
Total Gleason Score, *n* (ratio)	
6	3 (0.06)
7	14 (0.27)
> = 8	35 (0.67)
Biochemical recurrence (BCR), *n* (ratio)	
Yes	9 (0.17)
No	27 (0.52)
NA	16 (0.31)
Overall patient risk (OPR), *n* (ratio)	
Yes	23 (0.44)
No	27 (0.52)
NA	2 (0.04)
Follow-up (months), median (IQR)	41 (32–49)

*IQR* interquartile range, *NA* not available

### Delineation

**Fig. 2:**
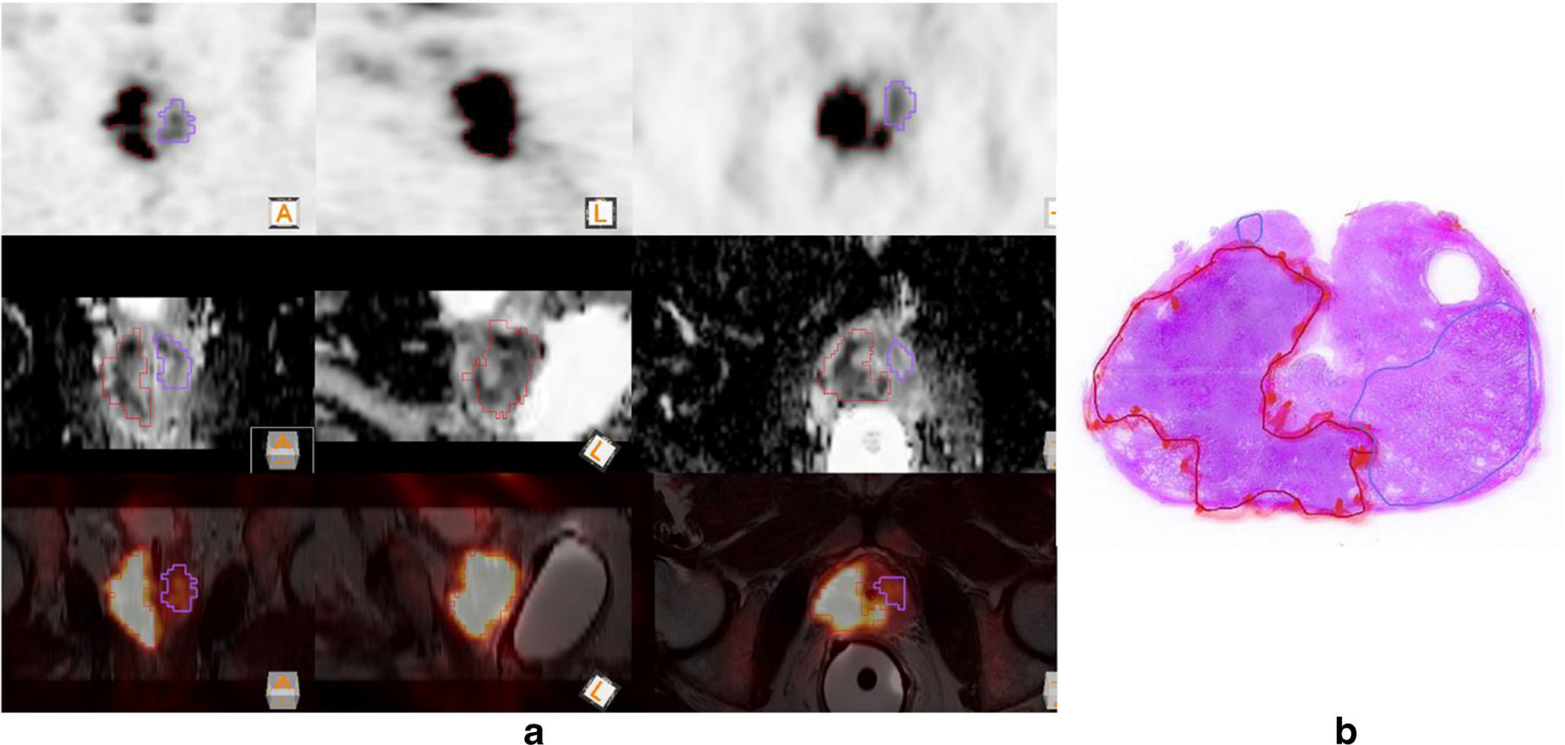
(**A**) Positron emission tomography/magnetic resonance imaging (PET/MRI) views of a prostate cancer patient with volumes of interests (VOIs) drawn over lesions with Gleason 4 (red) and high-grade pin (blue) patterns. Standard iso-count 3D VOIs were drawn over the [^68^Ga]Ga-PSMA-11 PET in the Hermes Hybrid 3D software. First row: [^68^Ga]Ga-PSMA-11 PET; second row: apparent diffusion coefficient (ADC) MRI; third row: fused [^68^Ga]Ga-PSMA-11 PET and transverse relaxation time-weighted (T2w) MRI images. Note that each image is represented in its own frame of reference, while the fused PET/MRI view is aligned to the frame of reference of the T2-weighted MRI. Hence, the cross-sections of the drawn VOIs look different on each view. (**B**) An example histopathological slice with the same color codes as in case of the PET/MRI views (red: Gleason 4, blue: high-grade pin)

Delineation and annotation of prostate lesions on PET/MR images were performed using the Hybrid 3D software ver. 4.0.0 (Hermes Medical Solutions, Stockholm, Sweden). Here, [^68^Ga]Ga-PSMA-11 PET and T2w as well as ADC MR images were viewed side-by-side with the annotated, whole-mount histopathological slices. Delineation was done over the [^68^Ga]Ga-PSMA-11 image using standard three-dimensional iso-count VOIs (Fig. 2). The initial lesion delineations were cross-examined and corrected manually—if required—as part of an independent review process performed by PET and MRI specialists. This step resulted in 121 lesions in total. An additional reference region was defined in the gluteus muscle to normalize the standard uptake value (SUV) of [^68^Ga]Ga-PSMA-11 and the T2w arbitrary voxel values to the mean of their respective reference background (26).

### Feature extraction

Each image was resampled to 2.0 × 2.0 × 2.0 uniform voxel resolution via ordinary Kriging interpolation [27, 28]. Radiomic features with “very strong” or “strong” consensus values as of the Imaging Biomarker Standardization Initiative (IBSI) guidelines were extracted from the 121 resampled [^68^Ga]Ga-PSMA-11, T2w and ADC lesions by the MUW Radiomics Engine (ver. 2.0) that was validated based on IBSI standards [29] (Supplement Table 1). Conventional standardized uptake values including SUX_max_, SUV_peak_, SUV_mean_, and SUV_TLG_ were merged with the extracted 442 radiomic features to compose a 446 long feature vector for each lesion. While total lesion glycolysis (TLG) is originally proposed for [^18^F]FDG, it was involved in our analysis as it characterized [^68^Ga]Ga-PSMA-11 accumulation in prostate lesions.

### Feature redundancy reduction

Feature redundancy ranking and reduction were done across the 446 features by covariance matrix analysis [19] where features were considered redundant with higher than 0.75 absolute Pearson correlation coefficient. This step resulted in keeping 80 features for further analysis.

### Reference standard

The respective whole-mount histopathology patterns of each delineated lesion were dichotomized as low (≤ Gleason 3, prostatic intraepithelial neoplasia (PIN), prostatitis, benign prostatic hyperplasia (BPH)) and high (> = Gleason 4) risk respectively. Furthermore, BCR and OPR reference values were established for each patient. BCR was defined when two consecutive PSA rose above 0.2 ng/ml. Follow-up was generally every 3 months for the first 2 years, then semiannually until the fifth year, then annually. Mean follow-up was 41 months. OPR was defined high, if BCR was positive or the node-stage (clinical or pathological) or the metastases-stage (clinical or pathological) were positive.

### Statistical analysis in [^68^Ga]Ga-PSMA-11

Area under the receiver operator characteristic curve (AUC) was calculated for conventional SUVs and the volume of each delineated lesion in the [^68^Ga]Ga-PSMA-11 image to estimate the performance of predicting low-vs-high lesion risk. This process included SUX_max_, SUV_peak_, SUV_TLG_, and lesion volume values.

### Cross-validation scheme

Monte Carlo (MC) cross-validation scheme was utilized to randomly assign training and validation roles to the 52 patients 1000-times. In each fold, five patients were selected for the validation role, while the remaining patients got the training role. This step was necessary to avoid mixing lesions for training and validation from the same patient. No repetitions were allowed during the generation of MC folds; thus, each of the 1000-fold configurations with their training-validation selections was unique.

### Machine learning scheme

Mixed ensemble learning scheme built on random forest classifiers (RF) was utilized to build models for predicting lesion LH, patient BCR as well as OPR (models denoted as M_LH_, M_BCR_ and M_OPR_ respectively) [26, 30, 31]. Nine RFs with various hyperparameters were configured for each of the three model schemes (Supplemental Table 2). The final prediction was provided by majority vote of the respective nine RFs. This approach was chosen to minimize hyperparameter bias and to increase predictive performance [32]. Furthermore, the average predictive score of the nine RFs represented a continuous value range between 0.0 and 1.0 reflecting on the prediction certainty of the mixed ensemble. Therefore, this value could be the subject of AUC analysis across MC folds.

### Lesion low-vs-high risk prediction

Training and validation lesion sets were generated as of the pre-generated MC scheme roles to train and validate the M_LH_ models in each MC fold. In order to keep model complexity minimal and to reduce the chance of overfitting, selection of the top five-ranking features was performed by *R*-squared ranking in the training dataset prior to establishing the M_LH_ lesion model per fold [33]. The same five features were then selected from the respective validation dataset to evaluate. Validation model performance was estimated via confusion matrix analytics across the predictions of the validation cases of the MC folds [26]. The M_LH_ scheme also underwent AUC analysis by evaluating the predictive performance of its averaged nine RF vote across the MC validation cases. Last, to estimate the effect of sham data in the M_LH_ model, confusion matrix analytics were also performed over randomly permutated labels across all MC folds [24, 34].

### Feature weighting

The importance of each feature in predicting lesion low-vs-high risk was determined by counting the occurrence of all selected features across the MC folds by the *R*-squared ranking approach.

### Patient biochemical recurrence and overall risk prediction

Patient risk models for predicting BCR and OPR were established (M_BCR_ and M_OPR_ respectively) analyzing the PSA, the enumerated clinical stage (Supplemental Table 3), and a composite M_LH_ score (CLH) per patient calculated by eq. 1.

1 $$ CLH=\sum \limits_{i=1}^k\frac{M_{LH}(i){v}_i}{V} $$

where *k* is the number of lesions in the given patient, *M*_*LH*_(*i*) is the predicted low-vs-high risk score of lesion *i* provided by the M_LH_ model of the given fold, *v*_*i*_ is the volume of lesion *i*, and $V={\sum}_{i=1}^k{v}_i$ is the sum of lesion volumes in the given patient.

Training and validation patient sets containing the above value triplets were generated as of the pre-generated MC scheme roles to train and validate the M_BCR_ and M_OPR_ models in each MC fold. In case a patient with validation role in the given fold had no BCR or OPR reference value available, it was excluded from the respective cross-validation of the given patient model.

To handle class imbalance, the training set underwent class imbalance correction by synthetic minority oversampling technique (SMOTE) [24, 35] for both the M_BCR_ and M_OPR_ training independently. Confusion matrix analytics were calculated across the validation set of all MC folds of the M_BCR_ and M_OPR_ model schemes. The same process was repeated by reference label permutations across the MC folds to estimate the effect of sham data. Both the M_BCR_ and M_OPR_ models underwent AUC analysis across the MC cross-validation folds.

## Results

### Patients

Of the 52 patients, 36 had BCR during follow-up and 50 had OPR information available at the time of conducting the study. At the time of radical prostatectomy, the average PSA was 7.5. The most common pathologic stages were stage 2 (*n* = 20, 38%) followed by 3b (*n* = 17, 33%) and 3a (*n* = 11, 21%). Total Gleason score occurrences were GS > =8 (*n* = 35, 67%) followed by GS 7 (*n* = 14, 27%) and GS = 6 (*n* = 3, 6%) (Table 1). The delineated 121 lesions represented a wide-range of benign and malign pathological alterations (Table 2). The most common high-risk pattern was associated to Gleason 4 (*n* = 50, 41%), followed by Gleason 3 (*n* = 17, 14%) and Gleason 5 (*n* = 11, 9%). Low-vs-high risk pattern regions were represented with balanced occurrences (*n* = 61-vs-60) (Table 2).

**Table 2:** Characteristics of the 121 delineated lesions in the 52 patients

Lesion characteristics (*n* = 121)	Value
Delineated lesions, *n* (ratio)	
Benign prostatic hyperplasia	20 (0.17)
Low grade PIN	16 (0.13)
High grade PIN	5 (0.04)
Prostatitis	2 (0.02)
Gleason 3	17 (0.14)
Gleason 4	50 (0.41)
Gleason 5	11 (0.09)
Lesion high-low risk pattern, *n* (ratio)	
High risk pattern	61 (0.504)
Low risk pattern	60 (0.496)

### Statistical analysis in [^68^Ga]Ga-PSMA-11

**Fig. 3:**
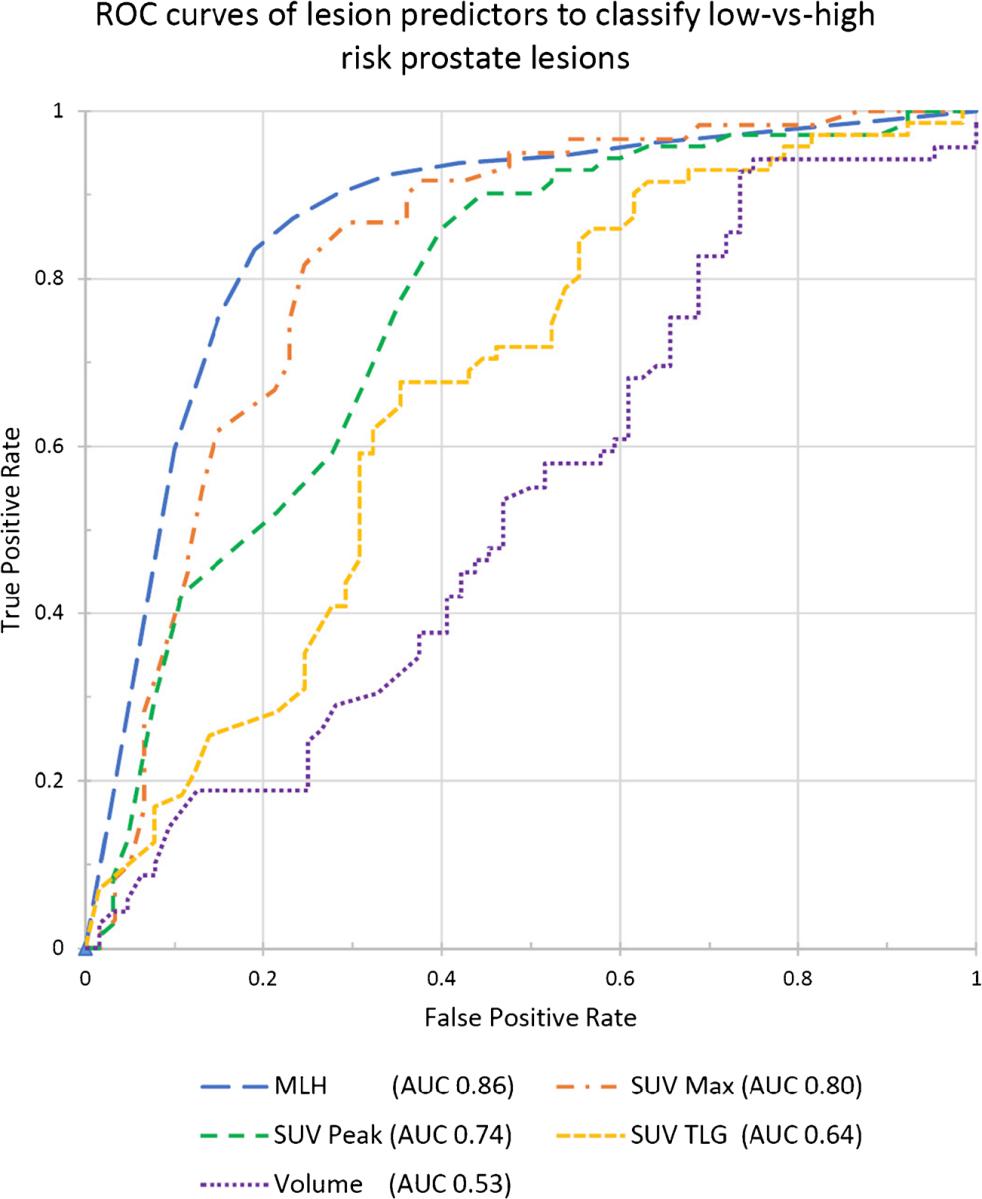
Area under the receiver operator characteristics curves (AUC) of conventional standardized uptake values (SUV) as well as lesion volume together with the machine learning low-vs-high lesion risk scores. Note that the M_LH_ AUC performance is a conservative estimate, as it is a Monte Carlo cross-validation AUC, while the SUV and volume curves were measured directly from the whole dataset

The AUC curves of SUV metrics were SUV_max_ 0.80, SUV_peak_ 0.74 and SUV_TLG_ 0.64. Lesion volume presented AUC of 0.53. In contrast, the low-vs-high lesion prediction model (M_LH_) demonstrated a cross-validation AUC of 0.86 which was the highest compared to conventional [^68^Ga]Ga-PSMA-11 values (Fig. 3).

### Lesion low-vs-high risk prediction

The M_LH_ model validation performance as per the MC cross-validation scheme yielded 71% sensitivity, 90% specificity, 88% positive predictive value, 75% negative predictive value, 81% accuracy, and 0.86 AUC. Sham data analysis revealed 0.52 AUC for permutated labels in the M_LH_ model.

### Feature weighting and distribution

**Fig. 4:**
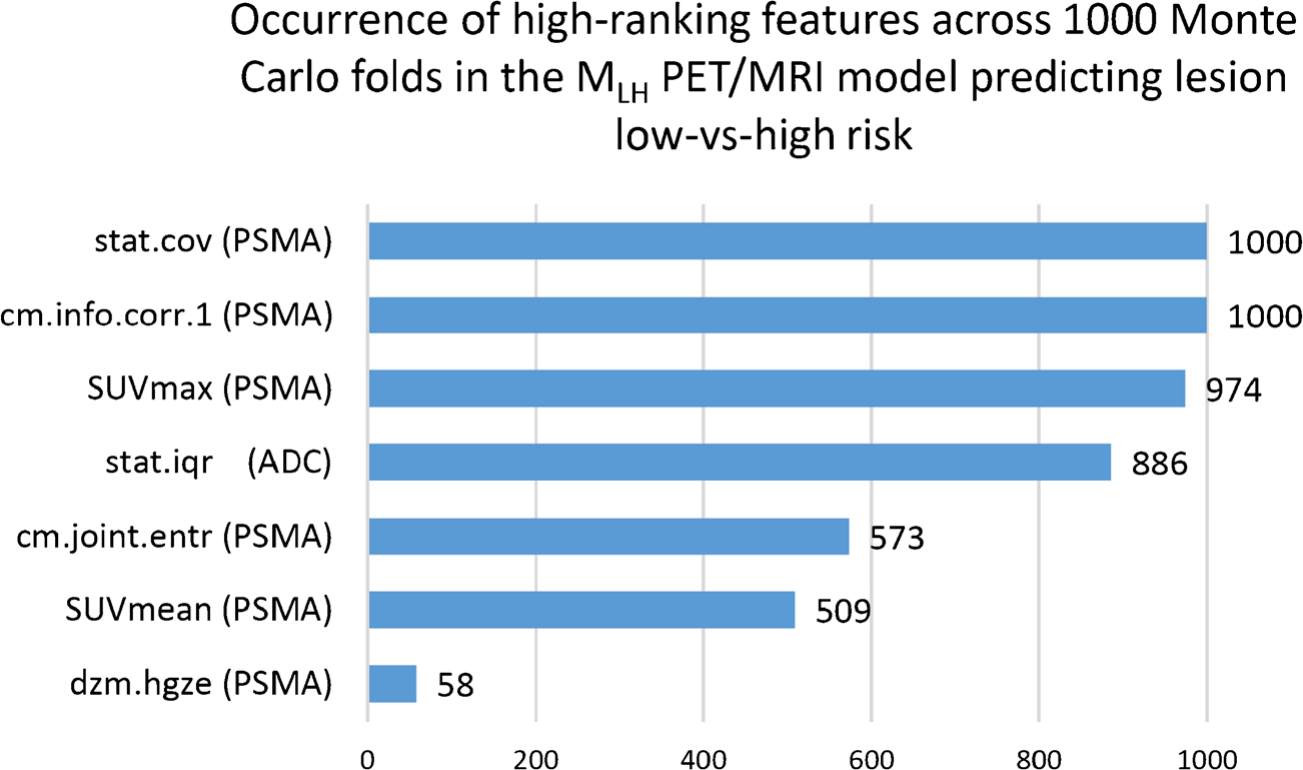
Occurrence of the highest ranked features across the 1000-fold Monte Carlo cross-validation scheme. PSMA—[^68^Ga]Ga-PSMA-11 positron emission tomography (PET); stat.cov: coefficient of variation; cm.info.corr.1—gray level co-occurrence matrix information correlation type 1; ADC—apparent diffusion coefficient; stat.iqr—interquartile range; cm.joint.entr—gray level co-occurrence matrix joint entropy; dzm.hgze—gray level distance zone matrix high gray zone emphasis

Overall seven features were identified as selected across the 1000 MC folds via the *R*-squared ranking method. Features that were always selected were coefficient of variation and gray level co-occurrence matrix (GLCM) information correlation type 1 from the [^68^Ga]Ga-PSMA-11 image (*n* = 1000). [^68^Ga]Ga-PSMA-11 SUV_max_ was the third mostly selected feature (*n* = 974) followed by the interquartile range of the ADC image (*n* = 886). GLCM joint entropy and SUV_mean_ were moderately prominent with (*n* = 573) and (*n* = 509) respectively in the [^68^Ga]Ga-PSMA-11 image. The lowest ranking feature (*n* = 58) was high gray zone emphasis in the [^68^Ga]Ga-PSMA-11 image (Fig. 4).

### Patient biochemical recurrence and overall risk prediction

**Fig. 5:**
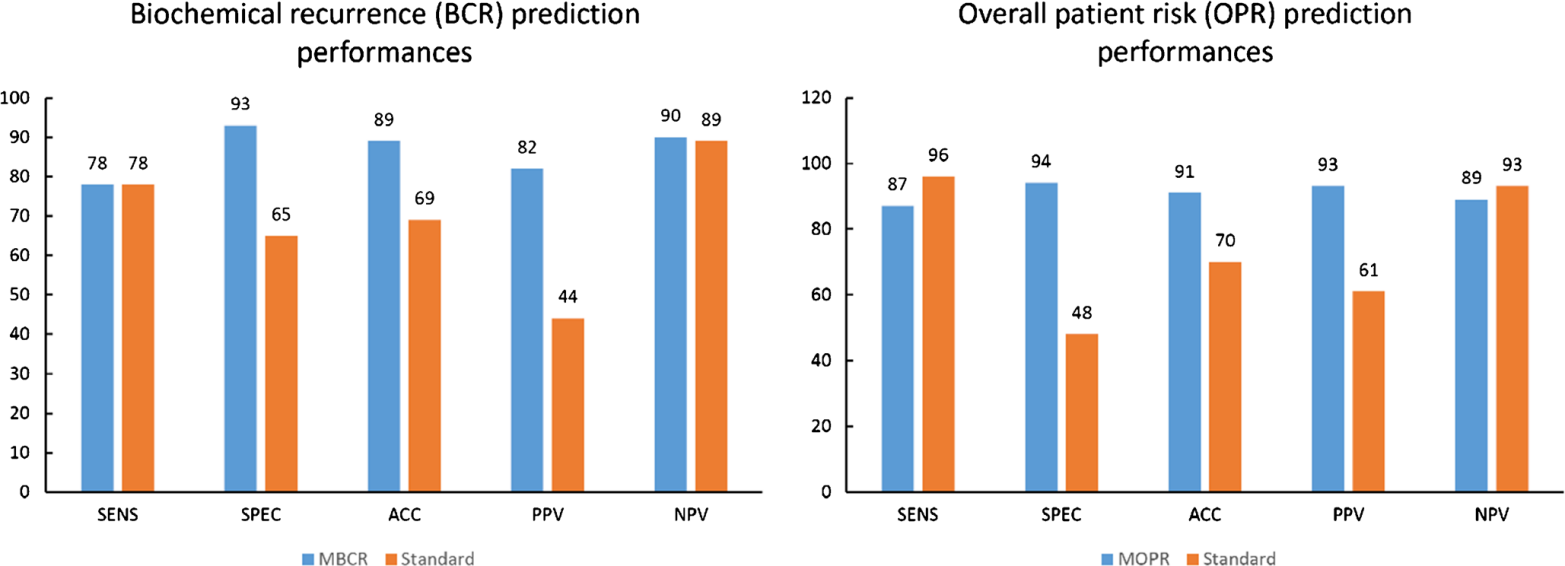
Left: validation performance estimations of predicting biochemical recurrence (BCR) by M_BCR_ and clinical standard models. Right: validation performance estimations of predicting overall patient risk (OPR) M_OPR_ and the clinical standard models. SENS—sensitivity; SPEC—specificity; ACC—accuracy; PPV—positive predictive value; NPV—negative predictive value. Confusion matrix values are in percentages. Note that standard risk estimator had a confusion analytics performance estimation in the whole dataset, as it is an established model, while the performance of M_BCR_ and M_OPR_ models was calculated through Monte Carlo cross-validation

The cross-validation performance revealed an average validation accuracy of 89% and 91% as well as AUC of 0.90 and 0.94 for the M_BCR_ and M_OPR_ patient models respectively. The M_OPR_ model outperformed the M_BCR_ model with 94% specificity, 93% positive predictive value, and with 87% sensitivity. The performance of M_OPR_ and M_BCR_ with sham data revealed 0.54 and 0.56 AUC respectively. See Fig. 5 for the detailed performance values of the M_BCR_ and M_OPR_ models.

## Discussion

In this study, we investigated the feasibility of predicting prostate lesion-specific low-vs-high risk built on PET/MRI radiomics and patient-specific biochemical recurrence as well as overall patient risk. We demonstrated excellent cross-validation performances for M_LH_ (AUC 0.86) as well as for M_BCR_ (AUC 0.90) and M_OPR_ (AUC 0.94). Based on the above approaches and our achieved model performances, we consider that our findings have important clinical implications in the field of primary prostate cancer risk assessment as they point towards the feasibility to estimate lesion and patient risks in vivo.

Next to establishing the above models with radiomics and machine learning, conventional [^68^Ga]Ga-PSMA-11 SUV and volume analysis were also conducted. This analysis revealed that SUV_max_ had the highest predictive power (AUC 0.80) to classify low-vs-high prostate lesions followed by SUV_peak_, and SUV_TLG_, while lesion volume had no significant predictive power (AUC 0.53). These findings are in line with previous analyses performed in PET/CT [24].

Feature ranking across our Monte Carlo folds demonstrated that [^68^Ga]Ga-PSMA-11 is the most important in vivo feature source to establish lesion risk prediction models compared to ADC and T2w MRI features. The highest-ranking [^68^Ga]Ga-PSMA-11 features were either simple statistical values such as the coefficient of variation and SUV_max_ or simple second-order textural ones such as information correlation from the GLCM feature category. Information correlation is a first-order GLCM feature reflecting on the information content (a.k.a. entropy) of voxel neighborhood connectivity occurrences; thus, it is a basic heterogeneity descriptor. This feature was previously also identified as highly robust across various PET imaging centers [36]. The feature ranking across MC folds identified SUV_peak_, SUV_TLG_, and volume as low-ranking; however, SUV_max_ was among the highest ranking ones. While the potential of PSMA SUV_max_ in characterizing prostate cancer had been presented [37, 38], Cysouw et al. concluded in a recent study that prostate risk in PSMA can be better characterized by textural parameters compared to SUVmax [24]. They utilized [^18^F]-DCFPyL PET/CT and reported 0.81 AUC to differentiate high (GS > = 8) and low-risk prostate cases. Our findings on the other hand demonstrate that conventional SUV parameters in combination with simple textural features can yield high-performing models in [^68^Ga]Ga-PSMA-11 PET/MRI to characterize prostate risk.

While no T2w feature was selected as high-ranking, ADC interquartile range (also referred to as “robust” value range) was selected as high-ranking. Prior studies focusing on ADC analysis to predict prostate lesion risk consistently identified ADC_min_, ADC_mean_ as well as ADC_median_ [20, 39] as highly predictive (AUC range 0.72–0.90). We consider that the above findings and ours describe the same phenomenon, namely, the strong predictive ability of simple ADC values without the need of incorporating second or higher-order radiomic features in the analysis. The above findings in prior reports demonstrate the predictive performance of PSMA PET and ADC MR images individually. Hence, we hypothesize that the high performance of our M_LH_ model is due to the fact that it combines both [^68^Ga]Ga-PSMA-11 PET and ADC MRI features in one model scheme.

Further to the above findings, we also established patient biochemical recurrence (M_BCR_) and overall patient risk (M_OPR_) models. In order to provide an in vivo score per patient in lieu of biopsy grades in these models, we created a CLH score which weighted each M_LH_ score per lesion with its respective volume in each patient. Since volume was identified as non-predictive to classify low-vs-high risk in prostate lesions (AUC 0.53), we assumed that the volume effect [40] in our high-ranking features was negligible, and thus, lesion volume was an independent value from our lesion M_LH_ scores. This assumption allowed us to utilize volume as a weight factor for each lesion M_LH_ score to compose the patient-specific CLH score. The resulted CLH score in combination with PSA and clinical stage values resulted in high-performing M_BCR_ and M_OPR_ models (0.90 and 0.94 cross-validation AUCs respectively). We assume that the accuracy performance increase of + 20% and + 21% in our M_BCR_ and M_OPR_ models compared to standard risk estimation are due to the following reasons: first, the clinical standard utilizes Gleason patterns from biopsy to describe lesion pattern risks in the prostate [41]. Biopsy is considered imperfect as it may not be able to describe the overall heterogeneity of the prostate lesions [19, 42]. In contrast, our CLH score could characterize whole prostate lesions in vivo. Second, the clinical standard categorizes the PSA, the Gleason, and the clinical stage values independently into three categories (low, medium, and high risk). In contrast, we incorporated PSA, clinical stage, and the CLH score without re-binning them, and thus, avoiding potential information loss. Third, the clinical standard score acts as a maximum filter across its pre-binned risk categories to estimate overall risk to the patient. In contrast, the random forest ensemble logics in our M_BCR_ and M_OPR_ models could describe more complex relationships among PSA, clinical stage, and our in vivo CLH score. Our results demonstrate that such relationships may be indeed present and that building on those relationships may lead to in vivo risk predictive models in prostate cancer patients with the potential to eliminate the need of biopsy sampling in the future.

This study had a number of limitations. First, it built on a single-center cohort; however, due to utilizing a pre-generated MC fold scheme for all training and validation processes, no training and validation samples were mixed in between the lesion and patient predictors. In addition, the utilized data preparation (redundancy reduction, feature ranking, and class imbalance correction) as well as training (mixed ensemble) and validation (1000-fold CV, sham data analysis) approaches minimized the chances of false discoveries. Second, due to the dual-tracer study design from which our images were taken, the [^68^Ga]Ga-PSMA-11 scans were not entirely exempt of [^18^F]FMC uptake remnants. Nevertheless, [^18^F]FMC can be regarded an irreversible tracer [43] and, thus, the [^18^F]FMC uptake in terms of tissue to lesion ratio is expected not to change until the [^68^Ga]Ga-PSMA-11 examination. Last, only patients with proven prostate cancer were included after radical prostatectomy. Nevertheless, this selection criterion was necessary to acquire stable ground truth for lesion labeling.

## Conclusions

This study demonstrates the feasibility of [^68^Ga]Ga-PSMA-11 PET/MRI in combination with radiomics and machine learning to non-invasively deliver both lesion characterization and risk prediction equally to preoperative invasive biopsy in patients with primary prostate cancer. Prospective multicentric studies are required to investigate the reproducibility and clinical utility of this approach.

## Supplementary Information

ESM 1 (DOCX 44 kb)

## Data Availability

The datasets generated and/or analyzed during the current study are available from the corresponding author on reasonable request.

## References

[1] Ferlay J, Colombet M, Soerjomataram I, Mathers C, Parkin DM, Piñeros M, et al. Estimating the global cancer incidence and mortality in 2018: GLOBOCAN sources and methods. Int J Cancer [Internet]. 2018;144(8):1941–53. 10.1002/ijc.31937. 10.1002/ijc.31937 30350310

[2] WCRF (2018). Protate cancer statistics.

[3] Mottet N, Bellmunt J, Bolla M, Briers E, Cumberbatch MG, De Santis M (2017). EAU-ESTRO-SIOG guidelines on prostate cancer. Part 1: screening, diagnosis, and local treatment with curative intent. Eur Urol [Internet].

[4] Hernandez DJ, Nielsen ME, Han M, Partin AW (2007). Contemporary evaluation of the D’Amico risk classification of prostate cancer. Urol Int.

[5] Fenton JJ, Weyrich MS, Durbin S, Liu Y, Bang H, Melnikow J (2018). Prostate-specific antigen-based screening for prostate cancer: a systematic evidence review for the U.S.

[6] Hatt M, Tixier F, Pierce L, Kinahan PE, Le Rest CC, Visvikis D. Characterization of PET/CT images using texture analysis: the past, the present… any future? Eur J Nucl Med Mol Imaging [Internet]. 2017;44(1):151–65. 10.1007/s00259-016-3427-0. 10.1007/s00259-016-3427-0 PMC5283691 27271051

[7] Preisser F, Bandini M, Marchioni M, Nazzani S, Tian Z, Pompe RS, et al. Extent of lymph node dissection improves survival in prostate cancer patients treated with radical prostatectomy without lymph node invasion. Prostate [Internet]. 2018;78(6):469–75. 10.1002/pros.23491. 10.1002/pros.23491 29460290

[8] Salmasi A, Faiena I, Wu J, Sisk AE, Sachveda A, Vandel JJ (2018). Radical prostatectomy then and now: surgical overtreatment of prostate cancer is declining from 2009 to 2016 at a tertiary referral center. Urol Oncol Semin Orig Investig [Internet].

[9] Chow K, Herrera P, Stuchbery R, Peters JS, Costello AJ, Hovens CM, et al. Late biochemical recurrence after radical prostatectomy is associated with a slower rate of progression. BJU Int [Internet]. 2019;123(6):976–84. 10.1111/bju.14556. 10.1111/bju.14556 30248237

[10] Galgano SJ, Valentin R, McConathy J (2018). Role of PET imaging for biochemical recurrence following primary treatment for prostate cancer. Transl Androl Urol [Internet].

[11] Herlemann A, Wenter V, Kretschmer A, Thierfelder KM, Bartenstein P, Faber C (2016). 68Ga-PSMA positron emission tomography/computed tomography provides accurate staging of lymph node regions prior to lymph node dissection in patients with prostate cancer. Eur Urol [Internet].

[12] Polanec SH, Andrzejewski P, Baltzer PAT, Helbich TH, Stiglbauer A, Georg D, et al. Multiparametric [11C]acetate positron emission tomography-magnetic resonance imaging in the assessment and staging of prostate cancer. Gelovani JG editor. PLoS One [Internet]. 2017;12(7):e0180790. 10.1371/journal.pone.0180790. 10.1371/journal.pone.0180790 PMC5515396 28719629

[13] Bouchelouche K, Choyke PL (2018). Advances in prostate-specific membrane antigen PET of prostate cancer. Curr Opin Oncol [Internet].

[14] Afshar-Oromieh A, Avtzi E, Giesel FL, Holland-Letz T, Linhart HG, Eder M, et al. The diagnostic value of PET/CT imaging with the 68Ga-labelled PSMA ligand HBED-CC in the diagnosis of recurrent prostate cancer. Eur J Nucl Med Mol Imaging [Internet]. 2015;42(2):197–209. 10.1007/s00259-014-2949-6. 10.1007/s00259-014-2949-6 PMC4315487 25411132

[15] Grubmüller B, Baltzer P, Hartenbach S, D’Andrea D, Helbich TH, Haug AR, et al. PSMA ligand PET/MRI for primary prostate cancer: staging performance and clinical impact. Clin Cancer Res [Internet]. 2018;24(24):6300–7. 10.1158/1078-0432.CCR-18-0768. 10.1158/1078-0432.CCR-18-0768 30139879

[16] Stone L. Predicting 68Ga-PSMA-PET–CT positivity for recurrent disease. Nat Rev Urol [Internet]. 2018;15:137. 10.1038/nrurol.2018.15. 10.1038/nrurol.2018.15 29434370

[17] Nair R, Porpiglia F, Zargar H. Re: MRI-targeted or standard biopsy for prostate-cancer diagnosis. Eur Urol [Internet]. 2018;74(4):524–5. 10.1056/NEJMoa1801993. 10.1016/j.eururo.2018.05.029 29886028

[18] Hartenbach M, Hartenbach S, Bechtloff W, Danz B, Kraft K, Klemenz B, et al. Combined PET/MRI improves diagnostic accuracy in patients with prostate cancer: a prospective diagnostic trial. Clin Cancer Res [Internet]. 2014;20(12):3244–53. 10.1158/1078-0432.CCR-13-2653. 10.1158/1078-0432.CCR-13-2653 24763613

[19] Gillies RJ, Kinahan PE, Hricak H. Radiomics: images are more than pictures, they are data. Radiology [Internet]. 2016;278(2):563–77. 10.1148/radiol.2015151169. 10.1148/radiol.2015151169 PMC4734157 26579733

[20] Manetta R, Palumbo P, Gianneramo C, Bruno F, Arrigoni F, Natella R (2019). Correlation between ADC values and Gleason score in evaluation of prostate cancer: multicentre experience and review of the literature. Gland Surg [Internet].

[21] Le MH, Chen J, Wang L, Wang Z, Liu W, Cheng K-T(T) (2017). Automated diagnosis of prostate cancer in multi-parametric MRI based on multimodal convolutional neural networks. Phys Med Biol [Internet].

[22] Liu C, Liu T, Zhang N, Liu Y, Li N, Du P, et al. 68Ga-PSMA-617 PET/CT: a promising new technique for predicting risk stratification and metastatic risk of prostate cancer patients. Eur J Nucl Med Mol Imaging [Internet]. 2018;45(11):1852–61. 10.1007/s00259-018-4037-9. 10.1007/s00259-018-4037-9 29717333

[23] Zamboglou C, Carles M, Fechter T, Kiefer S, Reichel K, Fassbender TF (2019). Radiomic features from PSMA PET for non-invasive intraprostatic tumor discrimination and characterization in patients with intermediate- and high-risk prostate cancer - a comparison study with histology reference. Theranostics [Internet].

[24] Cysouw MCF, Jansen BHE, van de Brug T, Oprea-Lager DE, Pfaehler E, de Vries BM, et al. Machine learning-based analysis of [18F]DCFPyL PET radiomics for risk stratification in primary prostate cancer. Eur J Nucl Med Mol Imaging [Internet]. 2020. 10.1007/s00259-020-04971-z. 10.1007/s00259-020-04971-z PMC7835295 32737518

[25] Epstein JI, Allsbrook WC, Amin MB, Egevad LL (2005). The 2005 International Society of Urological Pathology (ISUP) consensus conference on Gleason grading of prostatic carcinoma. Am J Surg Pathol [Internet].

[26] Papp L, Pötsch N, Grahovac M, Schmidbauer V, Woehrer A, Preusser M (2018). Glioma survival prediction with combined analysis of in vivo 11C-MET PET features, ex vivo features, and patient features by supervised machine learning. J Nucl Med.

[27] Phillips DL, Marks DG (1996). Spatial uncertainty analysis: propagation of interpolation errors in spatially distributed models. Ecol Model.

[28] Stytz MR, Parrott RW (1993). Using kriging for 3d medical imaging. Comput Med Imaging Graph.

[29] Zwanenburg A, Leger S, Vallières M, Löck S, Initiative for the IBS. Image biomarker standardisation initiative. arXiv [Internet]. 2016;(November). http://arxiv.org/abs/1612.07003

[30] Sagi O, Rokach L. Ensemble learning: a survey. Wiley Interdiscip Rev Data Min Knowl Discov [Internet]. 2018;8(4):1249. 10.1002/widm.1249.

[31] Parmar C, Grossmann P, Bussink J, Lambin P, Aerts HJWL (2015). Machine learning methods for quantitative radiomic biomarkers. Sci Rep.

[32] Feurer M, Hutter F. Hyperparameter optimization; 2019. p. 3–33. 10.1007/978-3-030-05318-5_1.

[33] van Timmeren JE, Leijenaar RTH, van Elmpt W, Reymen B, Oberije C, Monshouwer R (2017). Survival prediction of non-small cell lung cancer patients using radiomics analyses of cone-beam CT images. Radiother Oncol.

[34] Lacroix M, Frouin F, Dirand A-S, Nioche C, Orlhac F, Bernaudin J-F, et al. Correction for magnetic field inhomogeneities and normalization of voxel values are needed to better reveal the potential of MR radiomic features in lung cancer. Front Oncol [Internet]. 2020;10:43. 10.3389/fonc.2020.00043. 10.3389/fonc.2020.00043 PMC7006432 32083003

[35] Amin A, Anwar S, Adnan A, Nawaz M, Howard N, Qadir J (2016). Comparing oversampling techniques to handle the class imbalance problem: a customer churn prediction case study. IEEE Access.

[36] Papp L, Rausch I, Grahovac M, Hacker M, Beyer T (2019). Optimized feature extraction for radiomics analysis of 18F-FDG PET imaging. J Nucl Med.

[37] Woythal N, Arsenic R, Kempkensteffen C, Miller K, Janssen J-C, Huang K, et al. Immunohistochemical validation of PSMA expression measured by 68 Ga-PSMA PET/CT in primary prostate cancer. J Nucl Med [Internet]. 2018;59(2):238–43. 10.2967/jnumed.117.195172. 10.2967/jnumed.117.195172 28775203

[38] Bravaccini S, Puccetti M, Bocchini M, Ravaioli S, Celli M, Scarpi E (2018). PSMA expression: a potential ally for the pathologist in prostate cancer diagnosis. Sci Rep.

[39] Fehr D, Veeraraghavan H, Wibmer A, Gondo T, Matsumoto K, Vargas HA, et al. Automatic classification of prostate cancer Gleason scores from multiparametric magnetic resonance images. Proc Natl Acad Sci [Internet]. 2015;112(46):E6265–73. 10.1073/pnas.1505935112. 10.1073/pnas.1505935112 PMC4655555 26578786

[40] Traverso A, Kazmierski M, Zhovannik I, Welch M, Wee L, Jaffray D (2020). Machine learning helps identifying volume-confounding effects in radiomics. Phys Medica [Internet].

[41] D’Amico A. D’Amico Risk classification for Prostate Cancer [Internet]. https://www.mdcalc.com/damico-risk-classification-prostate-cancer

[42] Cook GJR, Siddique M, Taylor BP, Yip C, Chicklore S, Goh V (2014). Radiomics in PET: principles and applications. Clin Transl Imaging.

[43] Verwer EE, Oprea-Lager DE, van den Eertwegh AJM, van Moorselaar RJA, Windhorst AD, Schwarte LA, et al. Quantification of 18F-fluorocholine kinetics in patients with prostate cancer. J Nucl Med [Internet]. 2015;56(3):365–71. 10.2967/jnumed.114.148007. 10.2967/jnumed.114.148007 25678491

