# Supplementary material for: Supervised machine learning enables non-invasive lesion characterization in primary prostate cancer with [68Ga]Ga-PSMA-11 PET/MRI
Source: Eur J Nucl Med Mol Imaging. 2020 Dec 19;48(6):1795–805. doi: 10.1007/s00259-020-05140-y (PMC8113201; doi:10.1007/s00259-020-05140-y)
Supplement: Supplementary file 1 — (DOCX 44 kb) [file 259_2020_5140_MOESM1_ESM.docx]

**Supervised machine learning enables non-invasive lesion characterization in primary prostate cancer with [^68^Ga]Ga-PSMA-11 PET/MRI**

L. Papp^1^, C. P. Spielvogel^2,7^, B. Grubmüller^3^, M. Grahovac^2^, D. Krajnc^1^, B. Ecsedi^1^, R. A.M. Sareshgi^2^, D. Mohamad^2^, M. Hamboeck^2^, I. Rausch^1^, M. Mitterhauser^2,8^, W. Wadsak^2^, A. R. Haug^2,7^, L. Kenner^4,7^, P. Mazal^4^, M. Susani^4^, S. Hartenbach^5^, P. Baltzer^6^, T. H. Helbich^6^, G. Kramer^3^, S.F. Shariat^3^, T. Beyer^1^, M. Hartenbach^2**^; M. Hacker^2**^
 ^1^Medical University of Vienna, QIMP Team, Center for Medical Physics and Biomedical Engineering ^2^Medical University of Vienna, Department of Biomedical Imaging and Image-guided Therapy, Division of Nuclear Medicine
^3^Medical University of Vienna, Department of Urology
^4^Medical University of Vienna, Clinical Institute of Pathology

^5^HistoConsulting Inc., Ulm, Germany

^6^Medical University of Vienna, Department of Biomedical Imaging and Image-guided Therapy, Division of Common General and Pediatric Radiology

^7^Christian Doppler Laboratory for Applied Metabolomics

^8^Ludwig Boltzmann Institute Applied Diagnostics, Vienna, Austria

**shared senior authorship

Correspondence:

Marcus Hacker, MD

Department of Biomedical Imaging and Image-guided Therapy, Division of Nuclear Medicine

Medical University Vienna

Währinger Gürtel 18-20

1090 Vienna, Austria

marcus.hacker@meduniwien.ac.at

**SUPPLEMENTAL MATERIALS**

**TABLE 1**: IBSI reporting structure of the study as of the Imaging Biomarker Standardization Initiative (IBSI) guidelines (1)

| **Patient** | |
| --- | --- |
| Volume of Interest | [^68^Ga]Ga-PSMA-11 PET/MRI-positive lesions in prostate |
| Patient Preparation | As of clinicaltrials.gov NCT02659527 |
| Radiotracer | [^18^F]FMC |
|  | [^68^Ga]Ga-PSMA-11 |
| **Acquisition and Reconstruction** | |
| Protocol | As of clinicaltrials.gov NCT02659527 |
| Scanner type | Siemens Biograph 3T PET/MRI |
| [^18^F]FMC | - Static - 7 min bed position - 30 min after application - 2.08 x 2.08 x 2.03 mm voxel size |
| [^68^Ga]Ga-PSMA-11 | - Static (the last 10 min of dynamic scan) - 45 min after [^18^F]FMC (dual-tracer acquisition) - 2.08 x 2.08 x 2.03mm voxel size |
| T2w | - Static - TR - 750, TE - 109 and FA-100 - 0.9 x 0.9 x 0.9 mm voxel size |
| ADC | - Static - TR - 5300 and TE – 92 - 1.2 x 1.2 x 3.6 mm voxel size |
| TR – time of relaxation, TE – time of echo, FA – flip angle, ADC – Apparent Diffusion Coefficient | |
| **Image Co-registration** | |
| Software | Hermes Hybrid 3D ver 4.0 |
| Co-registration step 1 | Automated as of DICOM coordinate parameters |
| Co-registration step 2 | Optional in case of misalignments detected: ADC to T2w via an Automated Affine registration + manual correction if necessary |
| Co-registration step 3 | Optional in case of misalignments detected: All MRI sequences to [^68^Ga]Ga-PSMA-11 via automated rigid registration + manual correction if necessary |
| **Data conversion** | |
| Step 1 (all images) | Initial voxel values determined as of transforming the DICOM raw voxel values by the DICOM tags Rescale Scope (0028\|1053) and Rescale Intercept (0028\|1052). |
| Step 2 ([^68^Ga]Ga-PSMA-11, T2w) | Initial voxel values transformed to tumour-to-background ratio (TBR) by dividing all voxel values with the mean of the reference region drawn as a 9x9x9 cuboid VOI in the gluteus maximus region in each patient (drawn on T2w and copied to all other images). |
| **Segmentation** | |
| Software | Hermes Hybrid 3D ver 4.0 |
| VOI definition | Standard semi-automated iso-count 3D based on side-by-side viewing PET/MRI and respective annotated histopathological slices of the whole prostate. |
| Number of experts | 4+1 (4 nuclear medicine experts participated in independent delineations, followed by 1 senior nuclear medicine specialist cross-validation and if necessary, modification of first-round results) |
| Reference image | The image representing positive lesion (order of checking images for positive lesions: [^68^Ga]Ga-PSMA-11, T2w, ADC) |
| **Image / VOI interpolation** | |
| Method | Kriging interpolation in 3D (2), including nearest neighbors within distance of voxel size main diagonal + epsilon. |
| Grid | Align by center |
| Extrapolation beyond original image | Neighbor distance search calculated as original voxel size main diagonal + epsilon. Missing value: image minimum |
| Voxel dimensions | 2.0 x 2.0 x 2.0 mm |
| Partially masked voxels (VOI) | Taken if more than half of original voxel area included |
| **Discretization** | |
| Method | Fixed bin width, variable number of bins |
| Bin width | - [^68^Ga]Ga-PSMA-11, T2w: 0.05 (in TBR units) - ADC: 5 (in original units) |
| **Image biomarker computation / Parameters** | |
| Biomarker set | - Intensity features (21 per image): Minimum, Maximum, Mean, Sum, Variance, Skewness, Kurtosis, Median, 10th intensity percentile, 90th intensity percentile, Interquartile range, Range, Intensity-based mean absolute deviation, Intensity-based robust mean absolute deviation, Intensity-based median absolute deviation, Intensity-based coefficient of variation, Intensity-based quartile coefficient of dispersion, Energy, Root mean square intensity, Local intensity peak, Global intensity peak - Histogram features (19 per image): Mean discretised intensity, Discretised intensity variance, Discretised intensity skewness, (Excess) discretised intensity kurtosis, Median discretised intensity, Minimum discretised intensity, Maximum discretised intensity, Intensity histogram mode, Intensity histogram mean absolute deviation, Intensity histogram robust mean absolute deviation, Intensity histogram median absolute deviation, Intensity histogram coefficient of variation, Intensity histogram quartile coefficient of dispersion, Discretised intensity entropy, Discretised intensity uniformity, Maximum histogram gradient, Maximum histogram gradient intensity, Minimum histogram gradient, Minimum histogram gradient intensity - Intensity histogram features (6 per image): Volume at intensity fraction 10%, Volume at intensity fraction 90%, Intensity at volume fraction 10%, Intensity at volume fraction 90%, Volume fraction difference between intensity fractions, Intensity fraction difference between volume fractions - GLCM features (24 per image): Joint maximum, Joint average, Joint variance, Joint entropy, Difference Average, Difference variance, Difference entropy, Sum average, Sum variance, Sum entropy, Angular second moment, Contrast, Dissimilarity, Inverse difference, Normalised inverse difference, Inverse difference moment, Normalised inverse difference moment, Inverse variance, Correlation, Autocorrelation, Cluster shade, Cluster prominence, Information correlation 1, Information correlation 2 - Morphological features (13 per [^68^Ga]Ga-PSMA-11): Volume (mesh), Surface area (mesh), Surface to volume ratio, Compactness 1, Compactness 2, Spherical disproportion, Sphericity, Asphericity, Maximum 3D diameter, Volume density (axis-aligned bounding box), Area density (axis-aligned bounding box), Volume density (convex hull), Area density (convex hull) - Morphological features (3 per image): Moran’s I index, Geary’s C measure, Centre of mass shift - GLSZM features (16 per image): Small zone emphasis, Large zone emphasis, Low grey level zone emphasis, High grey level zone emphasis, Small zone low grey level emphasis, Small zone high grey level emphasis, Large zone low grey level emphasis, Large zone high grey level emphasis, Grey level non-uniformity, Normalised grey level non-uniformity, Zone size non-uniformity, Normalised zone size non-uniformity, Zone percentage, Grey level variance, Zone size variance, Zone size entropy - NGTDM features (5 per image): Coarseness, Contrast, Busyness, Complexity, Strength - GLRLM features (16 per image): Short runs emphasis, Long runs emphasis, Low grey level run emphasis, High grey level run emphasis, Short run low grey level emphasis, Short run high grey level emphasis, Long run low grey level emphasis, Long run high grey level emphasis, Grey level non-uniformity, Normalised grey level non-uniformity, Run length non-uniformity, Normalised run length non-uniformity, Run percentage, Grey level variance, Run length variance, Run entropy - NGLDM features (17 per image): Low dependence emphasis, High dependence emphasis, Low grey level count emphasis, High grey level count emphasis, Low dependence low grey level emphasis, Low dependence high grey level emphasis, High dependence low grey level emphasis, High dependence high grey level emphasis, Grey level non-uniformity, Normalised grey level non-uniformity, Dependence count non-uniformity, Normalised dependence count non-uniformity, Dependence count percentage, Grey level variance, Dependence count variance, Dependence count entropy, Dependence count energy - GLDZM features (16 per image): Small distance emphasis, Large distance emphasis, Low grey level zone emphasis, High grey level zone emphasis, Small distance low grey level emphasis, Small distance high grey level emphasis, Large distance low grey level emphasis, Large distance high grey level emphasis, Grey level non-uniformity, Normalised grey level non-uniformity, Zone distance non-uniformity, Normalised zone distance non-uniformity, Zone percentage, Grey level variance, Zone distance variance, Zone distance entropy |
| Software | MUW radiomics engine (3) ver. 2.0, developed and validated based on IBSI guidelines and reference datasets (1).  Software availability upon reasonable request from the corresponding author. |
| Distance weighting | No |
| CM symmetry | Symmetric |
| CM / ZM distance | Chebyshev distance 1 |
| CM / ZM aggregation | 3D, full-merging |
| Exclusion criteria | VOIs with less than 64 voxels were excluded from the analysis |

**Machine learning predictive models**

In order to build prediction models, nine random forest (4) machine learning approaches were employed in a mixed ensemble learning scheme (TABLE 2). The final model decision was performed by majority vote across the nine resulting RF models. This scheme was utilized to build lesion low-vs-high risk as well as patient BCR and OPR prediction models.

**TABLE 2**: Random forest (RF) algorithms settings of the nine RF models employed in the mixed ensemble learning scheme. KDE – Kernel density estimation (5). OOB – Outside of bag selection.

| **Model** | **RF-1** | **RF-2** | **RF-3** | **RF-4** | **RF-5** | **RF-6** | **RF-7** | **RF-8** | **RF-9** |
| --- | --- | --- | --- | --- | --- | --- | --- | --- | --- |
| Number of trees | 99 | 99 | 99 | 1000 | 1000 | 1000 | 1000 | 1000 | 1000 |
| Quality metric | gain | gain | gain | gain | gain | gain | gain | gain | gain |
| Maximum tree depth | 5 | 5 | 5 | 5 | 5 | 5 | 5 | 5 | 5 |
| Minimum samples at leaf | 10 | 10 | 10 | 5 | 5 | 10 | 5 | 5 | 10 |
| Feature selection | random | KDE | KDE | KDE | random | KDE | KDE | random | KDE |
| KDE attributes per split | NA | 5 | 5 | 5 | NA | 5 | 5 | NA | 5 |
| Random features per node ranking | 2 | NA | NA | NA | 3 | NA | NA | 3 | NA |
| Tree selection | NA | NA | NA | OOB | OOB | OOB | OOB | OOB | OOB |
| Number of selected trees | 99 | 99 | 99 | 99 | 99 | 99 | 99 | 99 | 99 |
| Bagging | equal | equal | equal | equal | equal | equal | equal | equal | equal |
| Bag fraction | 1.0 | 1.0 | 0.9 | 1.0 | 1.0 | 1.0 | 0.9 | 0.9 | 0.9 |

**Clinical stage enumeration**

**TABLE 3**: Enumeration approach to convert clinical stages to discrete numerical values to make it compatible for machine learning. Only clinical stages occurring in the dataset of this study are presented.

| Clinical stage | 2 | 2a | 2b | 2c | 3a | 3b | 4 |
| --- | --- | --- | --- | --- | --- | --- | --- |
| Enumerated clinical stage | 2 | 2.1 | 2.2 | 2.3 | 3.1 | 3.2 | 4 |

**References**

1. Zwanenburg A, Leger S, Vallières M, Löck S, Initiative for the IBS. Image biomarker standardisation initiative. arXiv [Internet]. 2016;(November). Available from: http://arxiv.org/abs/1612.07003

2. Stytz MR, Parrott RW. Using kriging for 3d medical imaging. Comput Med Imaging Graph. 1993;17(6):421–42.

3. Papp L, Rausch I, Grahovac M, Hacker M, Beyer T. Optimized feature extraction for radiomics analysis of 18 F-FDG-PET imaging. J Nucl Med [Internet]. 2018 Nov 2;jnumed.118.217612. Available from: http://jnm.snmjournals.org/lookup/doi/10.2967/jnumed.118.217612

4. Papp L, Spielvogel CP, Rausch I, Hacker M, Beyer T. Personalizing Medicine Through Hybrid Imaging and Medical Big Data Analysis. Front Phys [Internet]. 2018 Jun 7;6. Available from: https://www.frontiersin.org/article/10.3389/fphy.2018.00051/full

5. Geng X, Hu G. Unsupervised feature selection by kernel density estimation in wavelet-based spike sorting. Biomed Signal Process Control [Internet]. 2012 Mar 1 [cited 2017 Oct 17];7(2):112–7. Available from: http://www.sciencedirect.com/science/article/pii/S1746809411000279
